# Proteome data of *Anopheles stephensi* ovary using high-resolution mass spectrometry

**DOI:** 10.1016/j.dib.2018.08.066

**Published:** 2018-08-29

**Authors:** Gourav Dey, Ajeet Kumar Mohanty, Manish Kumar, Sreelakshmi K. Sreenivasamurthy, Arun H. Patil, T.S. Keshava Prasad, Ashwani Kumar

**Affiliations:** a Center for Systems Biology and Molecular Medicine, Yenepoya Research Center, Yenepoya (Deemed to be University), Mangalore 575018, India; b Institute of Bioinformatics, International Tech Park, Bangalore 560066, India; c Manipal Academy of Higher Education, Madhav Nagar, Manipal 576104, India; d ICMR-National Institute of Malaria Research, Field Unit, Campal, Panaji, Goa 403001, India; e School of Biotechnology, Kalinga Institute of Industrial Technology, Bhubaneswar, 751024, India

## Abstract

This article contains data on the proteins expressed in the ovaries of *Anopheles stephensi*, a major vector of malaria in India. Data acquisition was performed using a high-resolution Orbitrap-Velos mass spectrometer. The acquired MS/MS data was searched against *An. stephensi* protein database comprising of 11,789 sequences. Overall, 4407 proteins were identified, functional analysis was performed for the identified proteins and a protein-protein interaction map predicted. The data provided here is also related to a published article - “Integrating transcriptomics and proteomics data for accurate assembly and annotation of genomes” (Prasad et al., 2017) [1].

**Specifications Table**

**Table:** 

Subject area	Biology
More specific subject area	Vector biology
Type of data	Excel files, figures
How data was acquired	LTQ-Orbitrap Velos ETD mass spectrometer (Thermo Scientific, Bremen, Germany)
	Proteome Discoverer 2.1 and MASCOT search engine (Matrix Science, London, UK; version 2.2)
	Protein database *Anopheles stephensi* Liston (Indian strain) (www.VectorBase.org, release February 25, 2014)
Data format	Analyzed
Experimental factors	Ovaries were dissected from sugar fed mosquitoes and proteins extracted.
Experimental features	Proteome profiling of *Anopheles stephensi* ovaries
Data source location	Goa and Bangalore, India
Data accessibility	Raw mass spectrometric data is available from a web application (ProteomeXchange) Consortium
	(http://proteomecentral.proteomexchange.org/cgi/GetDataset?ID=PXD001128).
	Analyzed data is provided along with this article as excel sheets.
Related research article	Prasad et al. [1].

**Value of the data**

• The data tabulates proteins identified in the adult *An. stephensi* ovaries, which, plays an essential role in determining the fecundity of this important malaria vector.
• The data provides better understanding of the underlying physiology of *An. stephensi* females that can be manipulated to prevent the spread of malaria.
• The data provides potential protein-protein interactions among the proteins identified in the ovaries. Further study of these proteins can provide deeper insights in to the underlying physiological processes in female *An. stephensi*.
• The data provides a baseline platform for further studies on vector-pathogen interactions in female *An. stephensi* mosquitoes.

## 1 Data

**Fig. 1:**
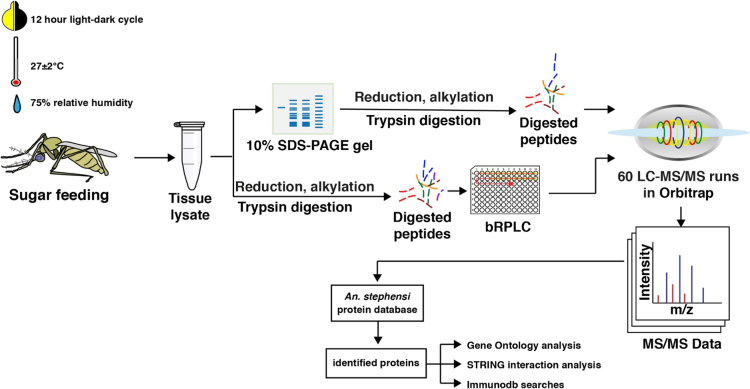
Pictorial representation of mosquito insectary conditions (light-dark cycle, temperature and humidity), sample processing steps, fractionation method used, and workflow of data analysis undertaken in the study.

To identify proteins expressed in the mosquito ovaries, we carried out high-resolution mass spectrometry-based proteomic analysis of ovaries dissected from sugar fed female *An. stephensi* Liston (Fig. 1). Extracted proteins were digested using trypsin followed by fractionation at protein level using SDS-PAGE. In addition, we also performed basic reverse phase liquid chromatograhy (bRPLC) fractionation at the peptide level. Each of these fractions were then analyzed on a high-resolution LTQ-Orbitrap Velos mass spectrometer and the acquired data searched against *An. stephensi* protein database. A total of 4407 were identified of which, 4284 proteins were identified with multiple PSMs. The complete list of proteins identified, and their corresponding peptides are provided in Supplementary Table S1 and S2.

## 2 Experimental design, materials and methods

### 2.1 Maintenance of mosquito colony

Continuous cyclic colony of *An. stephensi* mosquitoes were obtained from the insectary at ICMR-National Institute of Malaria Research, Field Station Goa. Colonies were maintained in a cycle of 12 hours in light and 12 hours in darkness, at 27±2 °C, 75% relative humidity. Adult mosquitoes were fed on 10% glucose solution.

### 2.2 Protein extraction

Altogether, 3,500 pairs of ovaries were dissected in 0.65% normal saline under dissecting microscope and homogenized in 200 µl of 4% SDS solution using a probe sonicator. Supernatant was collected after centrifugation of tissue lysate at 14,000 rpm for 10 minutes at 4 °C. Quantification of the extracted proteins were carried out according to Lowry׳s method (Bio-Rad DC Protein assay).

### 2.3 Fractionation

Proteins extracted from ovaries were fractionated as discussed in our previous studies [1], [2], [3]. For fractionation on gel, 300 µg of protein was resolved on a 10% SDS-PAGE, and 24 bands were excised. Excised bands were destained, reduced and alkylated followed by overnight digestion at 37°C using sequencing grade trypsin (Promega). Digested peptides were extracted and stored at -20 °C, until further use.

For fractionation at peptide level, 500 µg of protein were reduced and alkylated prior to trypsin digestion at 37 °C for 16 hours. Digested peptides were cleaned using Sepak C_18_ column and lyophilized prior to bRPLC fractionation. The lyophilized samples were reconstituted in bRPLC solvent A (10 mM triethyl ammonium bicarbonate (TEABC) in water at, pH 9.5), loaded on XBridge C_18_, 5 µm 250×4.6 mm column (Waters, Milford, MA) connected to Agilent 1100 series HPLC system. The peptide digest was resolved using a gradient of 5% to 100% solvent B (10 mM TEABC in Acetonitrile, pH 9.5) in 70 minutes. The total fractionation time was 60 minutes. Eluting peptides were collected in a 96 well plate and then concatenated into 36 fractions. Fractions were dried and reconstituted in 20 μl of 0.1% formic acid before mass spectrometric analysis [4].

### 2.4 Mass spectrometry analysis

The fractionated and cleaned peptides were analyzed on LTQ-Orbitrap Velos mass spectrometer (Thermo Scientific, Bremen, Germany) interfaced with Easy-nLCII (Thermo Scienific, Bremen, Germany). Peptides were enriched on a pre-column (2 cm, 5μ–100Ǻ), followed by separation on an analytical column (11 cm, 3μ–100Ǻ) packed in-house with magic AQ C_18_ material (Michrom Bioresources, Inc, Auburn, CA). The solvents used contained 0.1% aqueous formic acid as solvent A and 95% acetonitrile, 0.1% formic acid as solvent B. Solvent A was used to load peptides on to the trap column, followed by resolution on the analytical column using a gradient of 10–35% solvent B for 75 min at a constant flow rate of 0.25 μl/min. The spray voltage and heated capillary temperature were set to 2.0 kV and 220 °C. Data acquisition was carried out in a data dependent manner with a full scan in the range of m/z 350 to 2000. Orbitrap mass analyzer was used at both MS and MS/MS scans. Full MS scans were measured at a resolution 30,000 at m/z 400 and fifteen most intense precursors were selected for MS/MS fragmentation. Fragmentation was carried using higher-energy collisional dissociation (HCD) method and detected at a mass resolution of 15,000 at m/z 400. For full FTMS automatic gain control (AGC) was set to 1 million ions while FT MS/MS was set to 0.1 million ions with maximum accumulation time of 100 ms and 200 ms, respectively.

### 2.5 Functional categorization and prediction of interaction map

Categorization of the Gene Ontology terms for the identified proteins was performed by fetching information provided in the Panther database [5]. As Panther database has identifiers only for *An. gambiae*, which, is an ortholog of *An. stephensi*, we fetched the *An. gambiae* orthologs for the identified *An. stephensi* proteins using Biomart (version 0.7) [6] tool provided through VectorBase [7] (Supplementary Table S3). These *An. gambiae* identifiers were then used to fetch the Gene Ontology information. A predicted protein-protein interaction map of the identified proteins was then generated using STRING (Search Tool for the Retrieval Interacting Genes/Proteins) online tool (version 10.5) [8].

### 2.6 Data analysis

The mass spectrometry-derived data was searched against a database containing *An. stephensi* proteins along with known contaminants using Proteome Discoverer software, version 2.1 (Thermo Fischer Scientific, Bremen, Germany). The workflow consisted of Spectrum selector and SEQUEST and MASCOT search nodes. Trypsin was used as an enzyme allowing a single missed cleavage and 6 amino acids as the minimum peptide length. Search parameters also included carbamidomethylation of cysteine as static modifications and oxidation of methionine as a variable modification. Results were retrieved using a 1% false discovery rate (FDR) at the peptide level.

**Fig. 2:**
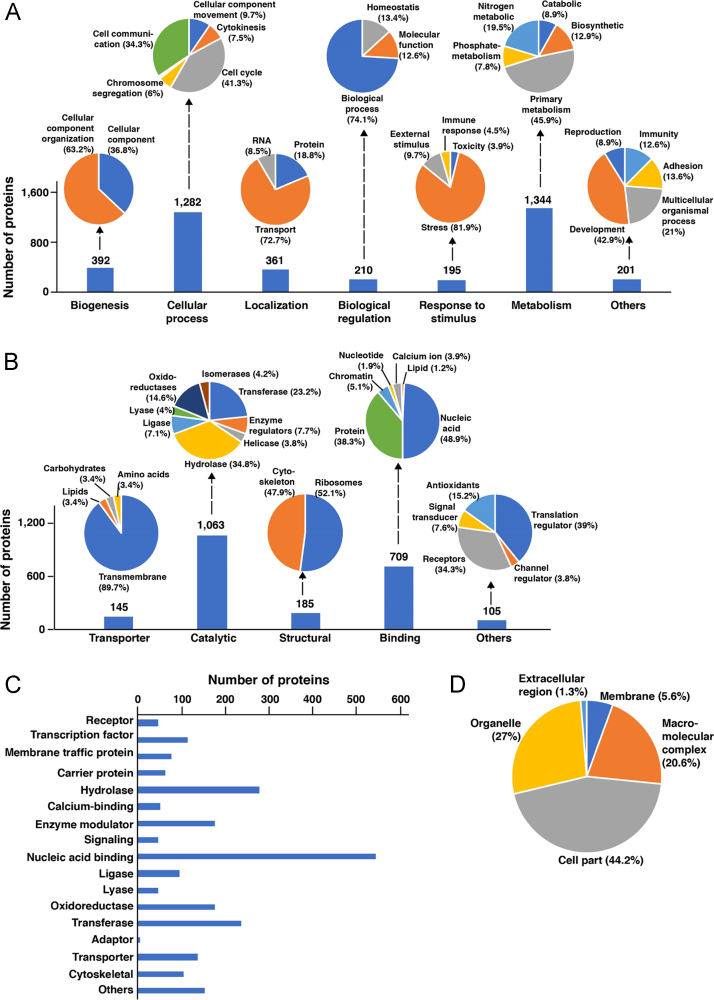
Functional analysis of the identified protein in *An. stephensi* ovaries. Gene Ontology information was fetched from PANTHER database. A) Biological processes, B) Molecular function, are represented in bar graph and major process and functions are further categorized into their sub-classes and represented as pie charts; C) Bar graph representation of Protein Class; D) Pie chart of Sub-cellular localization of the identified proteins.

**Fig. 3:**
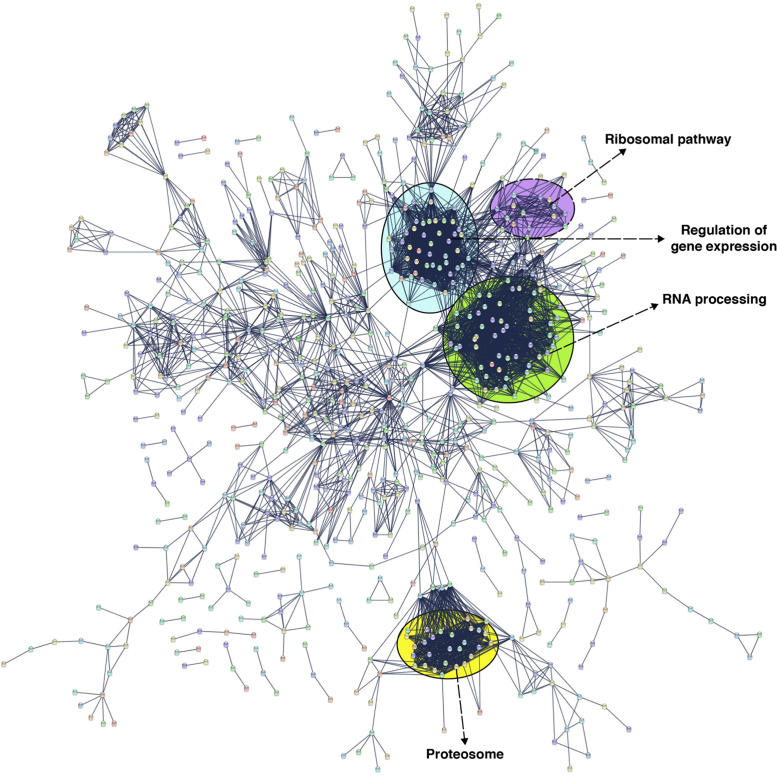
Predicted protein-protein interaction map of proteins identified in the ovary. Few of the identified proteins were found to cluster together into distinct clusters with associated roles in ribosomal pathway, regulation of gene expression, RNA processing and proteasomal pathway.

The proteins identified were annotated for their role in biological processes, molecular functions and their localization using Gene Ontology. Most of the identified proteins belonged to the protein class of nucleic acid binding proteins (23.1%), hydrolases (11.8%), transferases (10%) and oxidoreductases (7.5%). Functional annotations categorized the identified proteins to be associated with biological processes such as metabolism (33.6%), cellular processes (32%) and biogenesis (9.8%) (Fig. 2). We also generated a predictive protein-protein interaction map for the proteins identified in ovaries (Fig. 3, Supplementary Table S4). The interaction map showed distinctive clusters associated with ribosomal pathway, regulation of gene expression, RNA processing and proteasome pathways.

**Fig. 4:**
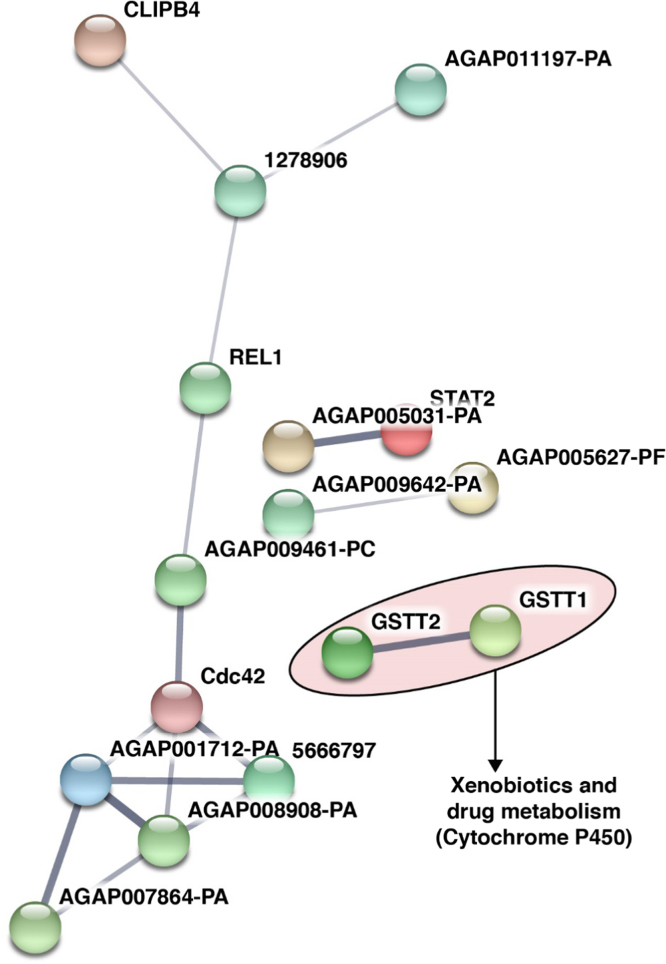
Predicted protein-protein interaction map of proteins identified in the *An. stephensi* ovary for which, their *An. gambiae* orthologs were found to have a role in vector-pathogen interactions.

**Fig. 5:**
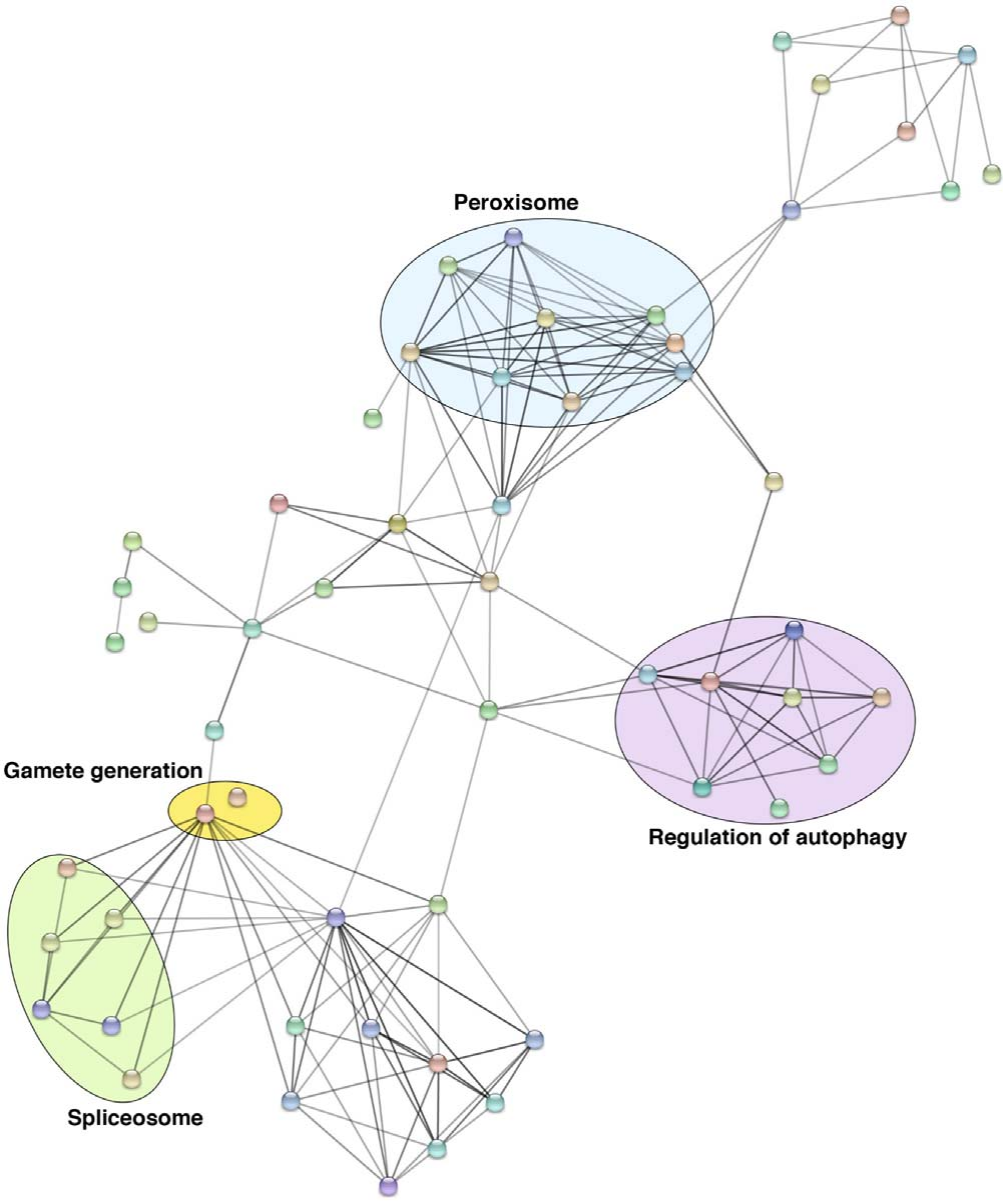
Predicted protein-protein interaction map of proteins and having a potential role in immunity (predicted by mapping to ImmunoDB database). Proteins associated with regulation of autophagy, spliceosome machinery, gamete generation and peroxisomes were predicted to be interacting among each other.

The potential role of certain genes in mosquitoes as agonist or antagonist to the development of *Plasmodium* parasite in the mosquito midgut and salivary glands has been studied through gene knockdown experiments [9]. We observed 30 of such proteins to be expressed in the *An. stephensi* ovaries with potential role in xenobiotics and drug metabolism that involves Cytochrome P450 (Fig. 4, Supplementary Table S5). However, these proteins were not restricted only to the ovaries and were also observed to be expressed in midgut, fatbody and salivary glands [1], [10], [11], [12]. The complete list of proteins identified in ovaries that mapped to the list of vector proteins with experimentally proven roles in parasite development is provided in (Supplementary Table S6). To further evaluate for related functions of the proteins identified in the ovaries, we mapped our data against Immunodb, which is a resource that provides a list of immune-related gene families. Seventy of the identified proteins were observed to have a potential role in mosquito vector immune responses. The list of proteins mapping to Immunodb is provided in (Supplementary Table S7). To evaluate the interaction network of the proteins mapping to Immunodb data, we generated a putative interacting map of the 70 proteins using STRING online tool (Fig. 5, Supplementary Table S8). Most of these proteins were found to be associated with peroxisome, regulation of autophagy, gamete generation and spliceosome.

The data presented here, thus facilitates further analysis and studies on *An. stephensi* by providing a baseline data.

## Appendix A Supplementary material

Supplementary material Supplementary Table S1: List of proteins identified in the ovaries of *An. stephensi* using high-resolution mass spectrometry-based proteomics approach.

.

Supplementary material Supplementary Table S2: List of peptides and their corresponding proteins that were identified in the ovaries of *An. stephensi* through high-resolution LC-MS/MS analysis.

.

Supplementary material Supplementary Table S3: List of proteins identified in *An. stephensi* (Indian strain) along with their orthologs in *An. stephensi* (SDA500 strain) and *An. gambiae*. Ortholog information was fetched from VectorBase using the provided Biomart tool.

.

Supplementary material Supplementary Table S4: List of proteins identified in ovaries of *An. stephensi* and predicted to be interacting among each other. Interaction map was constructed using STRING online tool with default search parameters**.**

.

Supplementary material Supplementary Table S5: Predicted protein-protein interactions of ovary proteins in *An. stephensi* (Indian strain) that may have a potential role in vector-parasite interactions.

.

Supplementary material Supplementary Table S6: List of proteins identified to be expressed in the ovary of *An. stephensi* and orthologs of which, were experimentally validated in *An. gambiae* for their role in *Plasmodium* development inside the vector mosquito.

.

Supplementary material Supplementary Table S7: List of proteins identified in *An. stephensi* ovaries that may have potential immune related functions according to the data obtained from Immunodb.

.

Supplementary material Supplementary Table S8: List of proteins expressed in *An. stephensi* ovary predicted to be associated with immune-related functions and predicted to be interacting with each other.
